# Digital Twins Use in Plastic Surgery: A Systematic Review

**DOI:** 10.3390/jcm13247861

**Published:** 2024-12-23

**Authors:** Ishith Seth, Bryan Lim, Phil Y. J. Lu, Yi Xie, Roberto Cuomo, Sally Kiu-Huen Ng, Warren M. Rozen, Foti Sofiadellis

**Affiliations:** 1 Department of Plastic Surgery, Peninsula Health, Melbourne, VIC 3199, Australia; 2 Plastic and Reconstructive Surgery, Department of Medicine, Surgery and Neuroscience, University of Siena, 53100 Siena, Italy; 3 Department of Plastic and Reconstructive Surgery, Austin Health, Heidelberg, VIC 3084, Australia

**Keywords:** digital twins, plastic surgery, personalised medicine, surgical precision, reconstructive surgery

## Abstract

**Background/Objectives:** Digital twin technology, initially developed for engineering and manufacturing, has entered healthcare. In plastic surgery, digital twins (DTs) have the potential to enhance surgical precision, personalise treatment plans, and improve patient outcomes. This systematic review aims to explore the current use of DTs in plastic surgery and evaluate their effectiveness, challenges, and future potential. **Methods:** A systematic review was conducted by searching PubMed, Scopus, Web of Science, and Embase databases from their infinity to October 2024. The search included terms related to digital twins and plastic surgery. Studies were included if they focused on applying DTs in reconstructive or cosmetic plastic surgery. Data extraction focused on study characteristics, technological aspects, outcomes, and limitations. **Results:** After 110 studies were selected for screening, 9 studies met the inclusion criteria, covering various areas of plastic surgery, such as breast reconstruction, craniofacial surgery, and microsurgery. DTs were primarily used in preoperative planning and intraoperative guidance, with reported improvements in surgical precision, complication rates, and patient satisfaction. However, challenges such as high costs, technical complexity, and the need for advanced imaging and computational tools were frequently noted. Limited research exists on using DTs in postoperative care and real-time monitoring. **Conclusions:** This systematic review highlights the potential of digital twins to revolutionise plastic surgery by providing personalised and precise surgical approaches. However, barriers such as cost, complexity, and ethical concerns must be addressed. Future research should focus on validating clinical outcomes through large-scale studies and developing soft tissue modelling and real-time monitoring capabilities.

## 1. Introduction

Digital twins are a sophisticated and innovative concept rooted in the engineering and manufacturing sectors, representing a real-time digital replica of a physical object, system, or process [1,2]. This rapidly advancing technology has applications across various fields, including healthcare [3,4]. In surgery, a digital twin provides a highly detailed virtual model that mirrors the patient’s anatomy and physiology [5], which is then dynamically updated with continuous data and real-time feedback loops from the patient [6,7]. This technology will be able to provide personalised treatment and has the potential to significantly enhance preoperative planning, intraoperative precision and decision-making, and postoperative clinical outcomes. Thus, the ability of digital twins to integrate patient-specific data with predictive modelling tools offers unprecedented opportunities for precision medicine and surgical planning [8,9].

Plastic surgery requires complex procedures considering individual anatomical variations and aesthetic results, making digital twins highly valuable. They provide detailed, patient-specific simulations that enhance surgical planning and execution, allowing for personalised approaches that improve outcomes, reduce intraoperative uncertainty, and minimise postoperative complications. In aesthetic plastic surgery, where patient satisfaction hinges on aesthetic results, digital twins offer a visual platform to simulate outcomes and align with patient expectations [10].

Conversely, recent advancements in machine learning and data processing could lower the barriers to integrating precision medicine with digital twinning [11]. Despite the apparent potential, integrating digital twin technology into plastic surgery is still nascent, and further targeted research is required. The current literature on digital twinning in plastic and reconstructive surgery needs to be more cohesive, with no systematic analysis critically examining digital twins’ effectiveness, challenges, and future possibilities in this specialty.

## 2. Materials and Methods

The methodology for this systematic review on digital twins in plastic surgery follows the PRISMA (Preferred Reporting Items for Systematic Reviews and Meta-Analyses) guidelines. The study was registered on PROSPERO (CRD420602094).

### 2.1. Search Strategy

A systematic search was conducted in multiple electronic databases, including PubMed, Scopus, Web of Science, and Embase, from their infinity to October 2024. The search was unrestricted by publication date to ensure the inclusion of early conceptual papers and recent advancements in the field. However, the language was limited to English. The initial search was performed in October 2024 and updated regularly until the final review process.

The search terms were constructed using keywords and Medical Subject Headings (MeSH) to ensure comprehensiveness. The search terms were organised into two main concepts: digital twins and plastic surgery. Examples of search terms included the following:

- (“digital twin” OR “virtual twin” OR “cyber-physical system” OR “computational model”);
- AND (“plastic surgery” OR “reconstructive surgery” OR “cosmetic surgery” OR “aesthetic surgery” OR “maxillofacial surgery” OR “faciomaxillary surgery”);
- AND (“simulation” OR “virtual model” OR “personalised surgery”).

Boolean operators combined the terms, and truncation was employed to capture various word forms. Additionally, the reference lists of all included studies and relevant reviews were manually searched to identify further studies that the database searches might have missed.

### 2.2. Inclusion Criteria

1. Study Type: Original research articles, randomised controlled trials, systematic reviews, clinical trials, cohort studies, and case series studies that describe the application of digital twin technology in plastic surgery. Both experimental and observational studies were considered.
2. Participants: Studies involving human subjects undergoing plastic, reconstructive, or cosmetic surgery where digital twin technology was used for preoperative planning, intraoperative decision-making, or postoperative follow-up.
3. Outcomes: Studies on clinical outcomes, patient satisfaction, surgical accuracy, complication rates, or cost-effectiveness associated with using digital twins in plastic surgery.
4. Technological Focus: Studies that explicitly mention using digital twins or comparable virtual models that simulate surgical procedures or predict outcomes in plastic surgery.
5. Language: Only studies published in English were considered.

### 2.3. Exclusion Criteria

1. Study Type: Editorials, commentaries, and conference abstracts were excluded unless they provided data from original research. Non-peer-reviewed articles were also excluded.
2. Technological Scope: Studies focusing on general simulation technologies without the specific use of digital twins, such as virtual reality or generic 3D modelling without real-time feedback mechanisms, were excluded.
3. Surgical Specialty: Studies focusing on other surgical fields outside plastic surgery, such as general, orthopaedic, or neurological surgery, were only allowed if the technology discussed directly applied to plastic surgery.
4. Duplicate Studies: Duplicate publications of the same study were excluded, although the most complete version of the study was included if data were presented in multiple papers.

### 2.4. Data Extraction

Two independent reviewers performed data extraction using a standardised data extraction form. Discrepancies between the reviewers were resolved through discussion or, if necessary, by consulting a third reviewer. The following data were extracted from each included study:

1. Study Characteristics: Author(s), year of publication, study design, and country of origin.
2. Participants: Number of participants, demographic information (e.g., age, gender), and the specific type of plastic surgery performed (e.g., breast reconstruction, facial surgery, limb reconstruction).
3. Digital Twin Technology: Description of the digital twin model used, including the type of software, hardware, data inputs (e.g., imaging, biomechanical data), and whether real-time feedback was incorporated.
4. Clinical Application: The stage of the surgical process where the digital twin was utilised (e.g., preoperative planning, intraoperative decision-making, postoperative monitoring).
5. Outcomes: The primary and secondary outcomes reported in each study, such as surgical precision, complication rates, patient satisfaction, length of hospital stay, and cost-effectiveness.
6. Study Limitations: The authors acknowledge limitations, such as small sample sizes, short follow-up periods, or technical challenges in implementing digital twin models.

### 2.5. Data Synthesis, Analysis, and Risk of Bias (Quality) Assessment

After data extraction (Table 1), a narrative synthesis was conducted to summarise the findings. The studies were categorised based on the type of digital twin application (e.g., reconstructive surgery, cosmetic surgery) and the clinical outcomes measured. Qualitative outcomes, such as patient satisfaction and surgeon experience with digital twins, were synthesised thematically. Studies were assessed for risk of bias using tools such as the AMSTAR2, JBI, and the Newcastle–Ottawa Scale for all included studies (Table 2).

## 3. Results

### 3.1. Study Characteristics

**Figure 1:**
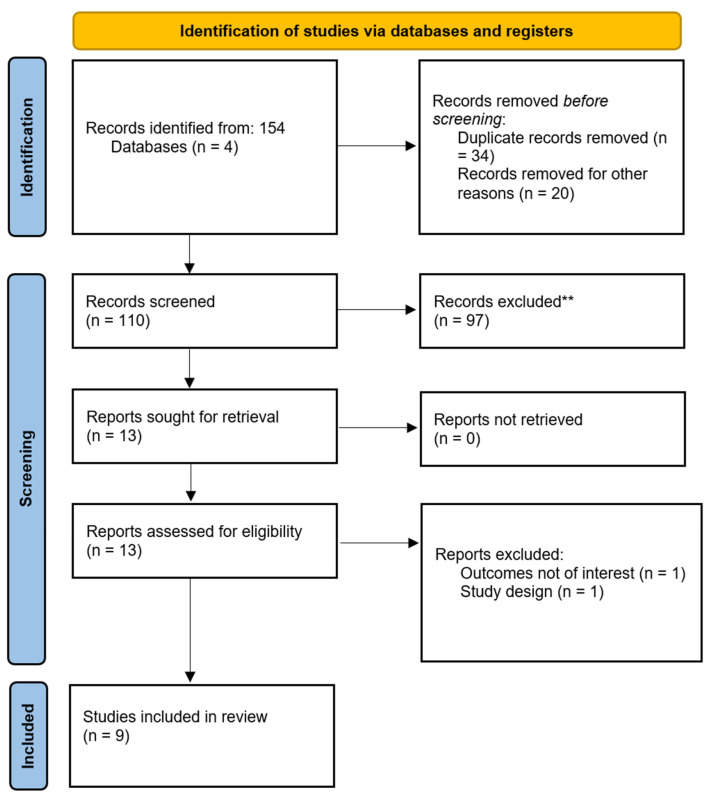
PRISMA flow diagram of selected studies.

A total of 110 studies were initially identified through a systematic search of PubMed, Scopus, Web of Science, and Embase databases, covering the time period from their infinity to October 2024. Figure 1 shows the PRISMA flow diagram of the included studies. Following title and abstract screening, studies were selected based on predefined inclusion criteria, which focused on research applying digital twin technology specifically to plastic, reconstructive, or cosmetic surgery, with relevance to preoperative planning, intraoperative guidance, or postoperative monitoring.

The selected studies demonstrated a range of applications of digital twins in preoperative planning and intraoperative guidance across plastic surgery. Systematic reviews such as those by Bosc et al. (2019) and Alkhayer et al. (2020) assessed the integration of augmented reality and virtual surgical planning for maxillofacial and orthognathic surgeries, respectively, reporting improved surgical precision and reduced intraoperative errors [12,15]. However, both reviews highlighted challenges related to the limited number of studies and variability in methodologies, indicating a moderate to high risk of bias due to inconsistencies and potential publication bias.

### 3.2. Clinical Outcomes

1. Surgical Precision: Multiple studies reported improvements in surgical precision through the use of digital twins. For example, Bosc et al. observed sub-millimetre accuracy in maxillofacial procedures when incorporating AR, and Alkhayer et al. reported maxillary and mandibular positioning errors within a 2 mm range for orthognathic surgeries. As highlighted by Ayoub and Pulijala (2019) [17], the use of virtual reality in maxillofacial surgery further supports the role of digital twin applications in achieving improved precision.
2. Patient Satisfaction and Outcomes: Patient satisfaction was consistently enhanced across studies that employed patient-specific surgical planning, as seen in rhinoplasty cases reported by Sobral et al. (2021) and Arias Gallo et al. (2023) [18,19]. Sobral et al. noted high patient satisfaction with improved aesthetic outcomes using accessible software tools for 3D virtual planning, while Arias Gallo et al. highlighted the utility of patient-specific surgical guides in improving alignment with preoperative goals.
3. Reduced Complication Rates: The systematic review and meta-analysis by Ohkuma et al. (2014) on breast reconstruction surgery provided quantitative evidence of reduced complication rates with the integration of computed tomographic angiography in preoperative planning, leading to a 13% decrease in flap-related complications and improved donor-site outcomes [20].

### 3.3. Limitations

While promising, the findings are limited by several constraints. Studies were generally small-scale, with many reporting small sample sizes, single-centre data, and limited follow-up durations, impacting the generalizability of results. For instance, the case series by Sobral et al. lacked a control group, restricting its ability to establish comparative effectiveness. Additionally, the high cost and technical complexity of digital twin technologies, as well as the need for advanced computational tools, were frequently cited as barriers to widespread adoption.

### 3.4. Risk of Bias

Quality assessment using tools such as AMSTAR 2, the JBI Critical Appraisal Tool, and the Newcastle–Ottawa Scale revealed a moderate to high risk of bias across studies, primarily due to issues with study heterogeneity, descriptive methodologies without controls, and limited patient-centred outcomes. While systematic reviews provided a robust overview, variability across included studies led to moderate risks of bias, as seen in the reviews by Bosc et al. and Alkhayer et al. The use of descriptive overviews, such as those by Ayoub and Pulijala, further contributed to reporting biases and limited evidence strength.

## 4. Discussion

Integrating digital twin technology in plastic surgery represents a promising frontier, offering substantial improvements in surgical precision, personalised treatment, and patient outcomes. This systematic review presents the status of the current literature on the application of digital twins in plastic surgery and highlights key advancements, challenges, and future directions. Digital twins, defined as real-time, dynamic virtual replicas of physical entities, are increasingly used in healthcare to simulate and predict clinical outcomes, optimise surgical procedures, and provide personalised treatment plans. This technology can revolutionise reconstructive and aesthetic surgeries in plastic surgery by enhancing preoperative planning, intraoperative guidance, and postoperative recovery [21,22].

Our review demonstrates that digital twin technology is primarily applied in preoperative planning and intraoperative guidance. For instance, digital twins provide patient-specific 3D breast anatomy modelling in breast reconstruction surgery, enabling surgeons to simulate different surgical approaches and predict postoperative outcomes [23]. This enhances surgical precision and allows for better-informed consent regarding their expected results, ultimately improving patient satisfaction. Furthermore, digital twins can simulate tissue deformation and predict responses to surgical interventions in the context of the patient’s unique anatomy [24,25].

Digital twins have been used to simulate bone and soft tissue reconstructions, enabling surgeons to plan complex procedures more accurately [26]. Integrating AI-driven predictive models into digital twins allows for real-time adjustments during surgery, thereby reducing the risk of complications and improving functional and aesthetic outcomes [27]. This is particularly important in procedures where millimetre-level precision is required, such as in cleft palate repairs or facial reconstruction following trauma. The use of digital twin technology in maxillofacial surgery represents the most advanced and extensively studied application within the surgical specialties, as evidenced by the reviewed studies. For instance, Bosc et al. (2019) successfully integrated augmented reality in maxillofacial procedures, achieving sub-millimetre precision with heads-up displays, enhancing surgical accuracy, and minimising intraoperative uncertainty [12]. Similarly, Alkhayer et al. (2020) reported a 2 mm error margin in virtual surgical planning for orthognathic surgery, showcasing significant improvements in maxillary and mandibular positioning accuracy [15]. Dubron et al. (2023) further reinforced the role of digital twins in orbital reconstructions, where preoperative AR-guided planning enabled precise implant placements despite variability in methods and equipment across studies [16].

In craniofacial surgery, Longeac et al. (2016) explored digital simulation for skill acquisition, emphasising its utility in surgical education and preoperative planning, albeit limited by small sample sizes [14]. Ayoub and Pulijala (2019) also highlighted the benefits of virtual reality in enhancing visualisation and improving surgical outcomes in oral and maxillofacial surgery [17]. However, the study lacked patient-centred outcome data. The consistent focus on hard tissue reconstructions in these studies highlights the suitability of digital twin technology for skeletal procedures, where rigid structures allow for highly accurate predictive modelling.

Despite the dominance of maxillofacial surgery studies, the findings provide a valuable foundation for expanding digital twin applications into other areas of plastic surgery, particularly reconstructive and aesthetic procedures. The demonstrated precision in craniofacial and orthognathic surgery, along with improved patient satisfaction seen in rhinoplasty studies (Sobral et al., 2021; Arias Gallo et al., 2023), underscores the potential for digital twins to address similar challenges in soft tissue modelling and microsurgical planning [18,19]. However, while digital twins have made significant strides in maxillofacial surgery, their limited application in broader plastic surgery fields, such as breast reconstruction and microsurgery, reflects a notable research gap. Future work should aim to build on these advancements, exploring the dynamic and deformable nature of soft tissues and expanding real-time feedback systems to enhance outcomes in complex plastic surgery procedures.

In reconstructive microsurgery, digital twins are utilised to model blood flow and tissue viability, helping surgeons predict the success of flap-based reconstructions [28]. These simulations provide critical information about the likelihood of graft survival and potential complications, enabling more informed decision-making during surgery. Using real-time feedback systems also allows for dynamic adjustments during the procedure, further optimising outcomes and reducing complication rates [29].

Overall, it is demonstrated that one of the critical benefits of digital twin technology in plastic surgery is its ability to provide highly personalised treatment plans. By incorporating patient-specific data such as imaging, biomechanical properties, and tissue characteristics, digital twins enable surgeons to tailor their approach to each patient. This level of personalisation is precious in plastic surgery, where achieving both functional and aesthetic outcomes is critical [30]. Additionally, digital twins offer significant advantages in terms of surgical precision. Simulating different surgical scenarios and visualising the potential outcomes before making an incision allows surgeons to plan their procedures more accurately. This is especially important in complex reconstructive surgeries, such as craniofacial reconstructions or bone reconstructions in the limbs, where even slight deviations can lead to suboptimal outcomes [31].

Beyond preoperative planning and patient consultation, digital twin virtual models can be used intraoperatively with real-time monitoring systems to dynamically adjust surgical approaches and techniques to optimise outcomes whilst minimising risks. Digital twin technology in plastic surgery enhances the understanding of surgical procedures through advanced simulation and analytics. Surgeons can explore various techniques and assess potential complications before surgery by visually representing the patient’s anatomy. This approach not only aids in refining skills but also supports training and education for surgical teams, leading to improved overall proficiency. Moreover, digital twins can facilitate collaboration among specialists by providing a shared, detailed model for discussion and strategy development, ensuring that diverse perspectives are considered in treatment planning. This collaborative environment is particularly beneficial for intricate cases where input from multiple disciplines can lead to innovative solutions. Ultimately, leveraging digital twin technology can transform how surgeries are approached, leading to more informed decision-making and enhanced patient safety.

Adopting digital twin technology, particularly virtual reality systems, presents specific risks and economic challenges. For VR system operators, potential risks include physical strain, such as fatigue, visual discomfort, and motion sickness, particularly with prolonged use. Additionally, the complexity of real-time simulations can lead to cognitive overload, impacting decision-making during preoperative planning or intraoperative guidance. Proper training and ergonomic enhancements are essential to mitigate these risks and ensure optimal system usability. From an economic perspective, implementing digital twin technology requires substantial investment in advanced imaging tools, computational infrastructure, and VR-compatible equipment. These costs and ongoing maintenance and training can limit accessibility, especially in resource-constrained settings. Economic justification depends on quantifiable benefits, such as improved surgical precision, reduced complications, and enhanced training outcomes. Robust cost-effectiveness studies are needed to evaluate the return on investment and identify pathways for more affordable adoption. Addressing these challenges will ensure that digital twin technology is both sustainable and beneficial in plastic and maxillofacial surgery.

Despite the promising potential of digital twin technology in plastic surgery, several challenges and limitations remain. One of the primary obstacles is the high cost of implementing digital twin systems. Developing accurate, real-time digital replicas requires advanced imaging technologies, sophisticated software, and significant computational power, all of which contribute to the high cost of adoption [31,32]. These costs may be prohibitive for many healthcare institutions, especially those in resource-limited settings.

Another significant challenge is the complexity of modelling soft tissue dynamics. While digital twins have been successfully used in orthopaedic and cardiovascular surgery, where the structures are relatively rigid and predictable, modelling the behaviour of soft tissues in plastic surgery presents additional challenges. Soft tissues, such as skin and fat, are highly variable and can respond unpredictably to surgical manipulation [33,34]. Developing accurate models that account for these variations is a complex task that requires further research and technological advancements. Ethical concerns also arise with digital twins, particularly regarding patient data privacy. Creating a digital replica of a patient raises questions about data ownership, consent, and security [35]. Ensuring that patient data is adequately protected and that patients fully understand how their data will be used is critical to addressing these concerns.

Additionally, while digital twins have shown promise in preoperative planning and intraoperative guidance, their use in postoperative care remains underexplored. Continuous monitoring and feedback through digital twins could improve postoperative outcomes by detecting complications early and enabling timely interventions. However, the integration of digital twins into postoperative care pathways is still in its infancy, and more research is needed to fully realise the potential benefits of this technology in the postoperative setting [12].

Several areas of future research and development are needed to overcome the current challenges and fully harness the potential of digital twin technology in plastic surgery. First, there is a need for more comprehensive and large-scale clinical trials to validate the effectiveness of digital twins in improving surgical outcomes. While the studies reviewed in this systematic review show promising results, most are limited by small sample sizes and short follow-up periods [13,14]. Second, advancements in machine learning and artificial intelligence could help address the challenges of modelling soft tissue dynamics. By incorporating AI-driven algorithms, digital twins could become more adaptive and capable of accurately predicting soft tissue behaviour [16]. These advancements would enable more precise simulations and improve the overall utility of digital twins in plastic surgery. Third, efforts should be made to reduce the cost of implementing digital twin technology. This could be achieved by developing more cost-effective imaging and computational tools and through collaborations between healthcare institutions and technology companies [17]. Additionally, training programs for surgeons on digital twin technology should be developed to facilitate its widespread adoption in clinical practice. Finally, the ethical implications of digital twins must be carefully considered. Clear data privacy, consent, and ownership guidelines should be established to protect patient rights [20]. As digital twin technology evolves, these ethical concerns must be addressed to build trust between patients and healthcare providers.

## 5. Conclusions

In conclusion, digital twin technology is promising to improve surgical precision, personalisation, and patient outcomes in plastic surgery. The ability to simulate and predict patient-specific outcomes before, during, and after surgery can revolutionise the field. However, challenges such as high costs, modelling complexities, and ethical concerns must be addressed before digital twins can be widely adopted in clinical practice. Future research should focus on validating the clinical benefits of digital twins, advancing soft tissue modelling, and developing cost-effective solutions for widespread implementation.

## Figures and Tables

**Table 1:** All included studies.

Author	Year	Study Design	Surgical Area	Technological Focus	Outcomes Reported	Limitations	Country
Bosc et al. [12]	2019	Systematic Review	Maxillofacial Surgery	Augmented reality (AR) in surgery	High precision (<1 mm error)	Limited to AR, small number of studies	France
Elnagar et al. [13]	2020	Descriptive Overview	Orthodontics and Orthognathic Surgery	Digital workflow for surgical planning	Improved surgical precision	Descriptive, not a clinical trial	USA
Longeac et al. [14]	2016	Original Research	Craniofacial Surgery	Digital simulation in surgical education	Improved skill acquisition	Limited by small sample size	USA
Alkhayer et al. [15]	2020	Systematic Review	Orthognathic Surgery	Virtual surgical planning	2 mm maxillary/mandibular accuracy	No cost analysis	Germany
Dubron et al. [16]	2023	Systematic Review	Orbital Reconstructions	AR in preoperative planning	Accurate placement of implants	2–3 mm higher error rates than traditional methods	Belgium
Ayoub and Pulijala [17]	2019	Descriptive Overview	Oral and Maxillofacial Surgery	Virtual reality (VR) in maxillofacial surgery	Improved accuracy in surgery	No patient outcome data	UK
Sobral et al. [18]	2021	Case Series	Rhinoplasty	3D virtual planning with Blender	Enhanced surgical precision, high patient satisfaction	Small sample size, no control group	Brazil
Arias Gallo et al. [19]	2023	Methodological Study	Rhinoplasty	Patient-specific surgical guides	Improved surgical outcomes with patient-specific guides	Small sample size, no long-term data	Spain
Ohkuma et al. [20]	2014	Systematic Review and Meta-Analysis	Breast Reconstruction	Virtual surgical planning using computed tomographic angiography (CTA)	Reduced flap-related complications, reduced donor-site morbidity, shorter operative times	Predominantly observational studies, generalizability affected by variability in techniques	Japan

**Table 2:** Quality assessment of studies on digital twin technology in plastic surgery.

**Author**	**Year**	**Study Design**	**Surgical Area**	**Quality Assessment Tool**	**Risk of Bias**	**Key Concerns**
Bosc et al. [12]	2019	Systematic Review	Maxillofacial Surgery	AMSTAR 2	Moderate to High	Limited number of studies; inconsistent data across included studies; potential publication bias.
Elnagar et al. [13]	2020	Descriptive Overview	Orthodontics and Orthognathic Surgery	JBI	High	The descriptive nature limits generalizability, lacks control or comparison groups, and creates a risk of reporting bias.
Longeac et al. [14]	2016	Original Research	Craniofacial Surgery	Newcastle–Ottawa Scale (NOS)	Moderate	Small sample size; limited follow-up; risk of selection bias due to single-centre study.
Alkhayer et al. [15]	2020	Systematic Review	Orthognathic Surgery	AMSTAR 2	Moderate	No cost analysis; variability in outcomes and measurement methods; potential for selection bias.
Dubron et al. [16]	2023	Systematic Review	Orbital Reconstructions	AMSTAR 2	Moderate	Variation in methods and equipment among studies; lack of patient-centred outcomes; moderate risk of bias due to inconsistent methodologies.
Ayoub and Pulijala [17]	2019	Descriptive Overview	Oral and Maxillofacial Surgery	JBI	High	No outcome data; lack of patient perspective; high potential for reporting and selection bias.
Sobral et al. [18]	2021	Case Series	Rhinoplasty	JBI	High	Small sample, no control group; high potential for selection and reporting bias due to lack of comparator or standardisation.
Arias Gallo et al. [19]	2023	Methodological Study	Rhinoplasty	Newcastle–Ottawa Scale (NOS)	Moderate	Small sample, no long-term data; risk of selection bias, limited scope and standardisation across methodology.
Ohkuma et al. [20]	2014	Systematic Review and Meta-Analysis	Breast Reconstruction	AMSTAR 2	Low to Moderate	Heterogeneity in study designs; lack of control over surgical techniques; overall high quality but limited by observational data nature.
